# The complete mitochondrial genomes of *Macrostylophora euteles* and *Citellophilus tesquorum sungaris* and the phylogenetics of known Siphonaptera mitogenomes

**DOI:** 10.3389/fvets.2025.1558328

**Published:** 2025-05-01

**Authors:** Mingna Duan, Yafang Liu, Jun Wu, Shuang Liu, Shaobo Tang, Dandan Jiang, Quanfu Zhang, Wei Gu, Xing Yang

**Affiliations:** ^1^Integrated Laboratory of Pathogenic Biology, College of Preclinical Medicine, Dali University, Dali, China; ^2^Department of Ophthalmology, Dali Bai Autonomous Prefecture People’s Hospital, Dali, China; ^3^School of Public Health, Dali University, Dali, China; ^4^Department of Gastroenterology, Clinical Medical College, The First Affiliated Hospital of Chengdu Medical College, Chengdu, China; ^5^Department of Infection, The First Affiliated Hospital of Dali University, Dali, China; ^6^The Key Laboratory of Infectious Diseases of Yunnan Provincial Education Department, Dali, China

**Keywords:** fleas, *Macrostylophora euteles*, *Citellophilus tesquorum sungaris*, mitochondrial genome, gene structure, phylogenetic

## Abstract

Fleas serve as hosts to a diverse array of pathogens, which present significant medical and veterinary concerns for human and livestock health. The mitochondrial genome (mtDNA) has long been regarded as a classical model in biogenetics and species evolution research. However, the availability of mitochondrial genome data for fleas remains scarce. In this study, we sequenced *Macrostylophora euteles* specimens collected from the Yunnan plague focus and *Citellophilus tesquorum sungaris* specimens from Jilin plague focus. The obtained sequences were compared to the sequences of 24 flea species retrieved from the NCBI database, focusing on base composition, evolution rates, nucleotide polymorphism and phylogenetic analysis. All fleas analyzed contained a total of 37 genes. Gene sequences exhibited remarkable stability, with no evidence of gene rearrangement. Additionally, the base composition demonstrated a pronounced AT bias. Results from both methodologies and across the two datasets consistently indicated strong monophyly for the superfamilies Ceratophylloidea and Pulicoidea, as well as for the family Pulicidae. In contrast, the superfamily Hystrichopsylloidea, along with the families Ceratophyllidae, Leptopsyllidae and Ctenophthalmidae, were identified as paraphyletic. This research provides valuable molecular data to support taxonomic and phylogenetic studies of fleas.

## 1 Introduction

Fleas are small, blood-feeding insects characterized by their smooth and tough body surface, typically ranging in color from brown to black-brown. These insects possess remarkable jumping abilities and hold medical significance. They belong to the phylum Arthropoda, class Insecta, and order Siphonaptera (23). To date, approximately 3,000 species and subspecies across 19 families have been identified globally (21). Fleas serve as primary vectors for the transmission of plague, making them critical indicators for identifying the early stages of plague epidemic. The majority of flea species primarily infest rodents, with only about 5% parasitizing birds (30). A wide range of zoonotic pathogens has been identified in fleas, including *Yersinia pestis*, *Rickettsia typhi*, *Rickettsia felis*, *Bartonella henselae*, *Bartonella quintana*, and *Francisella tularensis* (11, 14). Additionally, fleas function as intermediate hosts for *Dipylidium caninum* (41). Annually, these parasites and associated pathogens result in over $15 billion in global economic losses and have raised significant international public health concerns (36).

In recent years, there has been a notable rise in both emerging and re-emerging flea-borne diseases due to environmental changes and shifts in human behavior, posing significant challenges to public health (14). Consequently, the precise identification of flea species and systematic classification research are crucial for the prevention and control of these diseases. However, reaching a consensus on classification remains challenging, particularly concerning the number of families and included species (17). Traditional morphological identification methods require extensive professional experience and are often constrained by subtle morphological variations within species (27). With the advancements in genetic technology, molecular biological techniques have become increasingly prevalent for accurate identification and classification of fleas, thereby addressing some of the limitations associated with traditional morphology.

*Macrostylophora euteles* (Jordan and Rothschild, 1911 in wu (53)) is the first identified species within the genus *Macrostylophora* of the Ceratophyllidae family. This species primarily inhabits the provinces of Yunnan and Sichuan in China, with documented occurrences also reported in Myanmar and Thailand (10). *Citellophilus tesquorum sungaris* also referred to as *Citellophilus sungaris sungaris* (Jordan, 1929 in Wu (53)), a member of the *Citellophilus* genus within the same family, predominantly thrives in Inner Mongolia, Jilin and Harbin regions of China, as well as in Russia and Mongolia (34). Notably, it serves as a key vector in marmot- or ground squirrel-associated plague foci in northeastern China (13). As primary vectors of plague, fleas provide an early warning signal for assessing plague outbreaks and play a crucial role in maintaining the natural focus of plague and the continuity of the biological community (39).

As a classical model for structural, functional and evolutionary studies, the mitochondrial genome holds significant value in the fields of biogenetics and species evolution (18, 25). To address the scarcity of mitochondrial genome resources for fleas and to provide molecular insights into flea prevention and control, as well as to enhance our understanding of phylogenetic relationships, we conducted a study involving the sequencing of the complete mitogenomes of *M. euteles* and *C. tesquorum sungaris*. We also analyzed their genetic characteristics and phylogenetic relationships.

## 2 Methods

### 2.1 Sample collection and DNA extraction

The adult fleas of the *M. euteles* were collected from the body surface of *Callosciurus erythraeus* in Xiangyun County, Yunnan Province (25° 33′N, 100° 77′E), China. Meanwhile, specimens of *C. tesquorum sungaris* were obtained from *Cricetulus barabensis* in Qian’an County, Jilin Province (44° 99′N, 124° 12′E), China. All flea specimens were preserved in EP tubes containing 95% ethanol, transported to the laboratory for identification by professionals based on morphological characteristics (53), and subsequently stored at −20°C for future use. Prior to DNA extraction, the specimens underwent cleaning with sterile double distilled water followed by drying on sterile filter paper. Genomic DNA was then separated using a DNA Extraction Kit (TIANGEN BIOTECH, Beijing, China) according to the manufacturer’s instructions.

### 2.2 Mitochondrial sequencing and assembly

Sequencing was conducted using second-generation sequencing (NGS) on the Illumina NovaSeq platform to construct gene libraries, perform paired-end sequencing of these libraries to obtain raw gene fragment data, and conduct preliminary assembly of quality-controlled clean data to generate all possible scaffolds. Mitochondrial genomes from closely related species with available sequences were used as reference datasets, and the resulting assembled sequences were compared using BLAST for *de novo* assembly. After generating raw data in FASTQ format, we calculated the number of reads, total base count, and GC content. Data filtering criteria primarily involved utilizing AdapterRemoval version 2 (43) software for removing 3’end adapters, thereby eliminating contamination and ensuring high-quality data. High-quality data underwent *de novo* assembly using SPAdes v3.15.5 (38), followed by correction using Pilon v1.19 (50) to obtain the final mitochondrial sequence.

### 2.3 Gene annotation and data analysis

The mitogenome assembly was performed using A5-miseq v20150522 (8). The mitochondrial genomes of *M. euteles* and *C. tesquorum sungaris* were annotated via the MITOS WebServer^1^ (3). Circular maps of the mitogenomes were generated using the CGView Server^2^ (46). Relative synonymous codon usage (RSCU) was evaluated with CodonW v1.4.2, while nucleotide composition analysis was conducted using DNA star v11.1. The skewness of the relative base composition was calculated using the formulas GC-skew = (G − C)/(G + C) and AT-skew = (A − T)/(A + T) (37). The DnaSP v6.12.3 (42) was utilized to conduct analyses of evolutionary rates and nucleotide diversity. The former was evaluated through the ratios of non-synonymous (Ka) to synonymous (Ks) substitutions (Ka/Ks), while the latter was assessed using a sliding window approach with a window size of 100 and a step size of 25.

### 2.4 Phylogenetic relationships analysis

To elucidate the phylogenetic relationships within the order Siphonaptera, mitochondrial genome sequences from 26 flea species were employed to construct phylogenetic trees. This dataset included two newly sequenced species from this study and an additional 24 species obtained from the NCBI database (Table 1). *Boreus elegans* (HQ696579) was designated as outgroup, and 13 protein-coding genes (PCGs) were utilized for the phylogenetic analysis of these 27 species. The amino acid and nucleotide sequences were aligned using MAFFT v7.520 (20), followed by trimming of ambiguous regions with Gblocks v0.91b (47). Subsequently, the sequences were concatenated in PhyloSuite v1.2.3 (54) to produce two distinct datasets: one comprising the amino acid sequence dataset (PCGaa) and the other consisting of the nucleotide sequence dataset (PCGnt).

**Table 1:** Taxonomic information and GenBank accession numbers of 26 mitogenomes of Siphonaptera were selected for characterization and phylogenetic analysis.

Superfamily	Family	Genus	Species	Accession No.	References
Ceratophylloidea	Ceratophyllidae	*Ceratophyllus*	*Ceratophyllus wui*	NC_040301	(48)
		*Ceratophyllus*	*Monopsyllus anisus*	NC_073017	(28)
		*Citellophilus*	*Citellophilus sungaris sungaris*	PP418872	This study
		*Jellisonia*	*Jellisonia amadoi*	NC_022710	(4)
		*Macrostylophora*	*Macrostylophora euteles*	OR774969	This study
		*Nosopsyllus*	*Nosopsyllus laeviceps*	PP838812	(12)
	Leptopsyllidae	*Frontopsylla*	*Frontopsylla diqingensis*	NC_085276	Unpublished
		*Paradoxopsyllus*	*Paradoxopsyllus custodis*	OQ627398	(5)
		*Frontopsylla*	*Frontopsylla spadix*	NC_073018	(29)
		*Leptopsylla*	*Leptopsylla segnis*	NC_072691	(28)
Hystrichopsylloidea	Ctenophthalmidae	*Ctenophthalmus*	*Ctenophthalmus yunnanus*	NC_085277	Unpublished
		*Ctenophthalmus*	*Ctenophthalmus quadratus*	NC_072692	(6)
		*Stenischia*	*Stenischia montanis yunlongensis*	OR780663	(5)
		*Stenischia*	*Stenischia montanis*	PP990561	Unpublished
		*Stenischia*	*Stenischia humilis*	NC_073020	(6)
		*Stenoponia*	*Stenoponia polyspina*	OR834393	(26)
		*Neopsylla*	*Neopsylla specialis*	NC_073019	(29)
	Hystrichopsyllidae	*Hystrichopsylla*	*Hystrichopsylla weida qinlingensis*	NC_042380	(49)
	Stivaliidae	*Aviostivalius*	*Aviostivalius klossi*	OR774970	Unpublished
Pulicoidea	Pulicidae	*Pulex*	*Pulex irritans*	NC_063709	(56)
		*Ctenocephalides*	*Ctenocephalides orientis*	NC_073009	(2)
		*Ctenocephalides*	*Ctenocephalides felis felis*	MW420044	(57)
		*Ctenocephalides*	*Ctenocephalides canis*	NC_063710	(56)
		*Ctenocephalides*	*Ctenocephalides felis*	NC_049858	(9)
		*Xenopsylla*	*Xenopsylla cheopis*	MW310242	(51)
Vermipsylloidea	Vermipsyllidae	*Dorcadia*	*Dorcadia ioffi*	NC_036066	(55)

Phylogenetic relationships were evaluated using maximum likelihood (ML) analysis with IQ-TREE v2.3.6 (35) and Bayesian inference (BI) with MrBayes v3.2.7 (40). For the ML analysis, the optimal model for the nucleotide dataset of PCGs was determined to be GTR + F + I + G4, while the most appropriate model for the amino acid dataset of PCGs was identified as mtART + F + I + G4 using ModelFinder (19), with a bootstrap value of 1,000 replicates. The BI analysis involved four simultaneous Markov Chain Monte Carlo (MCMC) runs over two million generations, sampling every 1,000 generations, with the first 25% of data discarded as burn-in. The final phylogenetic tree was visualized and refined utilizing FigTree v1.4.4^3^ and the Interactive Tree of Life (iTOL) v6.9.1 (22).

## 3 Results

### 3.1 Mitogenome structure and nucleotide composition

In this study, we conducted a thorough analysis of the mitochondrial genomes of two newly sequenced flea species alongside 24 fleas previously published in the NCBI database. In the newly sequenced species *M. euteles* and *C. tesquorum sungaris*, a total of 37 genes were identified, comprising 13 PCGs, 22 transfer RNA (tRNA) genes, and 2 ribosomal RNA (rRNA) genes (Supplementary Figure S1). Of these 37 genes, 23 are situated on the heavy strand (H strand), while the remaining 14 are located on the light strand (L strand) (Supplementary Table S1). The mitochondrial genomes of *M. euteles* and *C. tesquorum sungaris* measure 16,027 and 15,345 bp in length, respectively. The average mitochondrial genome length for Siphonaptera is 16,963 bp, with *Ctenocephalides orientis* exhibiting the longest genome at 22,189 bp and *Stenoponia polyspina* having the shortest at merely 14,933 bp (Table 2). Using *cox1* as a reference, we observed that the mitochondrial gene arrangements in 26 flea species are consistent with the putative ancestral mitochondrial genome arrangement of *Drosophila yakuba* and previously sequenced Mecoptera species, providing no evidence of gene rearrangement.

**Table 2:** The base features of Siphonaptera mitochondrial genomes.

Species	Mitochondrial genome base content				PCGs base content				tRNAs base content		rrnL		rrnS	
	Length(bp)	A + T(%)	AT-skew	GC-skew	Length(bp)	A + T(%)	AT-skew	GC-skew	length	A + T(%)	length	A + T(%)	length	A + T(%)
*Ceratophyllus wui*	18,081	76.71	−0.0166	−0.1833	11,057	76.87	−0.1473	0.0091	1,432	79.61	1,239	80.79	780	81.03
*Ceratophyllus anisus*	15,875	78.54	−0.0220	−0.2311	11,137	76.39	−0.1464	0.0161	1,433	80.04	1,218	80.95	779	81.00
*Citellophilus tesquorum sungaris*	15,345	78.07	−0.0292	−0.2175	11,030	76.42	−0.1481	0.0263	1,429	79.78	1,248	81.17	781	80.92
*Jellisonia amadoi*	17,031	79.17	−0.0203	−0.2597	11,119	76.71	−0.1511	0.0039	1,431	79.66	1,293	81.67	787	80.05
*Macrostylophora euteles*	16,027	77.59	−0.0079	−0.2682	11,114	74.91	−0.1434	0.0100	1,431	80.36	1,287	81.12	780	78.97
*Nosopsyllus laeviceps*	16,533	78.10	−0.0287	−0.1653	11,143	78.17	−0.1430	0.0440	1,433	80.60	1,196	81.69	774	81.14
*Frontopsylla diqingensis*	15,878	79.33	−0.0349	−0.2143	11,143	77.42	−0.1338	0.0133	1,439	80.19	1,281	82.98	786	81.30
*Paradoxopsyllus custodis*	15,375	76.79	−0.0077	−0.2589	11,111	74.82	−0.1430	0.0044	1,432	80.31	1,290	81.09	780	79.10
*Frontopsylla spadix*	15,085	78.83	−0.0362	−0.2146	11,144	77.47	−0.1336	0.0129	1,439	80.19	1,281	82.98	786	81.42
*Leptopsylla segnis*	15,785	78.89	0.0236	0.2477	11,138	77.08	−0.1440	−0.0026	1,420	79.58	1,274	81.95	780	81.28
*Ctenophthalmus yunnanus*	15,801	79.36	−0.0159	−0.2277	11,118	77.65	−0.1356	0.0197	1,415	80.07	1,251	81.85	780	80.90
*Ctenophthalmus quadratus*	15,938	79.45	−0.0135	−0.2253	11,126	77.75	−0.1397	0.0292	1,405	80.28	1,250	81.52	783	80.72
*Stenischia montanis yunlongensis*	15,651	77.29	−0.0116	−0.2373	11,118	74.99	−0.1463	0.0052	1,425	79.09	1,281	80.95	784	81.25
*Stenischia montanis*	15,889	77.54	−0.0125	−0.2373	11,124	75.02	−0.1458	0.0064	1,425	79.09	1,281	80.95	784	81.25
*Stenischia humilis*	15,617	78.00	−0.0110	−0.2382	11,118	75.90	−0.1431	0.0199	1,424	79.71	1,266	81.52	785	81.02
*Stenoponia polyspina*	14,933	78.81	−0.0044	−0.2312	11,124	77.80	−0.1283	0.0018	1,405	79.64	1,299	82.14	782	81.33
*Neopsylla specialis*	16,820	77.27	0.0001	−0.2512	11,142	74.84	−0.1534	−0.0060	1,408	78.84	1,262	80.43	791	79.01
*Hystrichopsylla weida qinlingensis*	17,173	80.59	−0.0297	−0.2210	11,129	78.01	−0.1557	0.0191	1,424	80.20	1,224	82.11	786	80.66
*Pulex irritans*	20,337	80.02	−0.0271	−0.1461	11,095	78.07	−0.1464	0.0388	1,439	79.78	1,294	82.77	793	82.09
*Ctenocephalides orientis*	22,189	83.21	−0.0511	−0.2579	11,082	80.02	−0.1373	0.0100	1,425	81.12	1,303	83.42	799	82.73
*Ctenocephalides felis felis*	20,911	82.88	−0.0442	−0.2371	11,094	80.19	−0.1423	0.0419	1,415	80.78	1,302	83.72	785	82.55
*Ctenocephalides canis*	15,609	78.52	−0.0171	−0.1816	11,082	79.25	−0.1473	0.0395	1,412	80.59	1,300	83.46	798	81.83
*Ctenocephalides felis*	20,873	83.13	−0.0444	−0.2294	11,093	80.20	−0.1431	0.0429	1,416	80.72	1,299	83.76	787	82.72
*Xenopsylla cheopis*	18,902	82.83	−0.0105	−0.2213	11,064	80.31	−0.1195	0.0386	1,426	80.79	1,312	83.16	797	82.94
*Aviostivalius klossi bispiniformis*	16,593	79.04	−0.0008	−0.1698	11,059	77.20	−0.1174	0.0219	1,436	80.15	1,317	82.23	791	79.90
*Dorcadia ioffi*	16,785	80.71	−0.0063	−0.1975	11,134	78.10	−0.1419	0.0420	1,436	79.39	1,302	82.03	782	80.31

The mitogenomes of fleas demonstrate a pronounced AT bias, with AT content varying from 76.71% in *Ceratophyllus wui* to 83.21% in *C. orientis*, resulting in an average AT content of 79.26%. The AT-skew ranged from −0.0511 in *C. orientis* to 0.0236 in *Leptopsylla segnis*, while GC-skew spanned from −0.2682 in *M. euteles* to 0.2477 in *L. segnis*. Notably, *L. segnis* was the only species exhibiting a positive GC-skew, whereas all other species showed negative values (Table 2).

### 3.2 PCGs, evolution rate and nucleotide diversity

**Figure 1:**
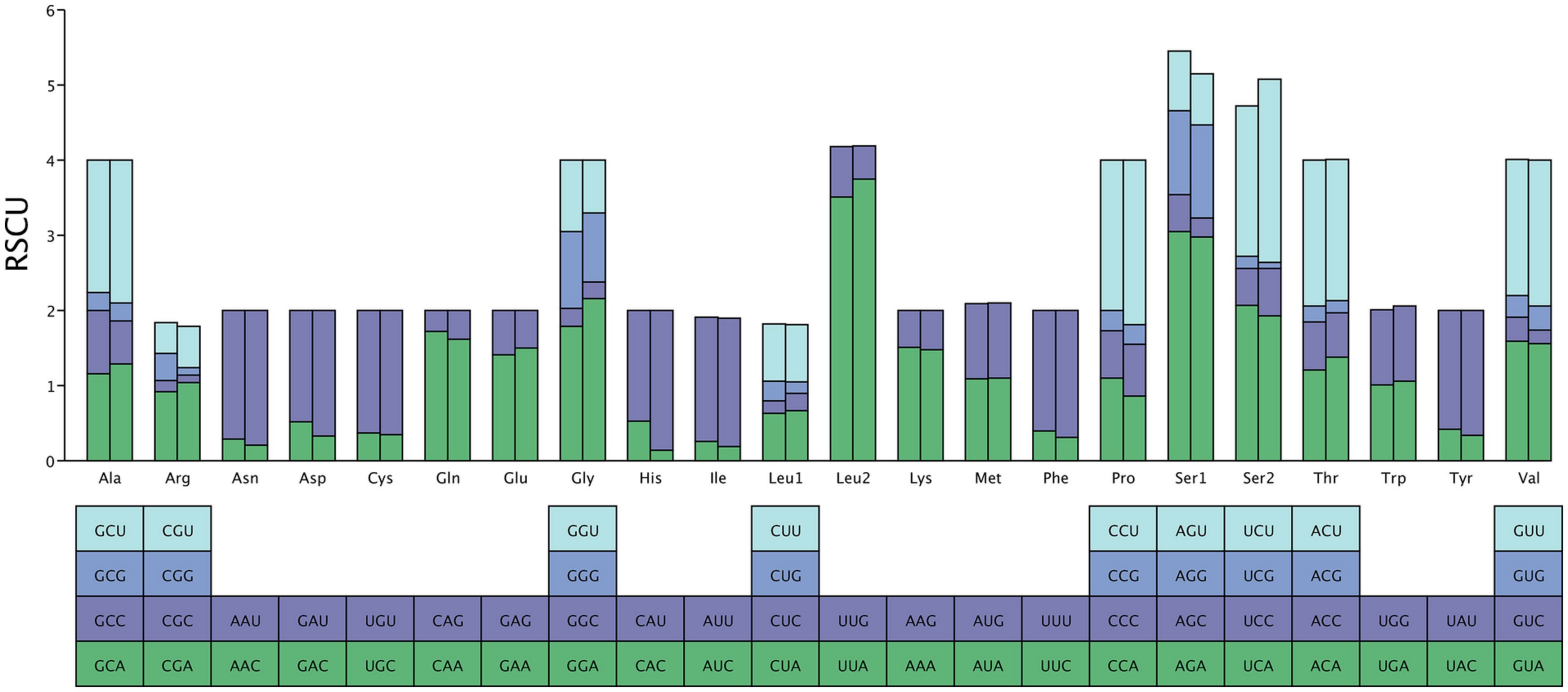
The relative codon usage of two newly sequenced mitochondrial genome, with *M. euteles* on the left and *C. tesquorum sungaris* on the right.

**Figure 2:**
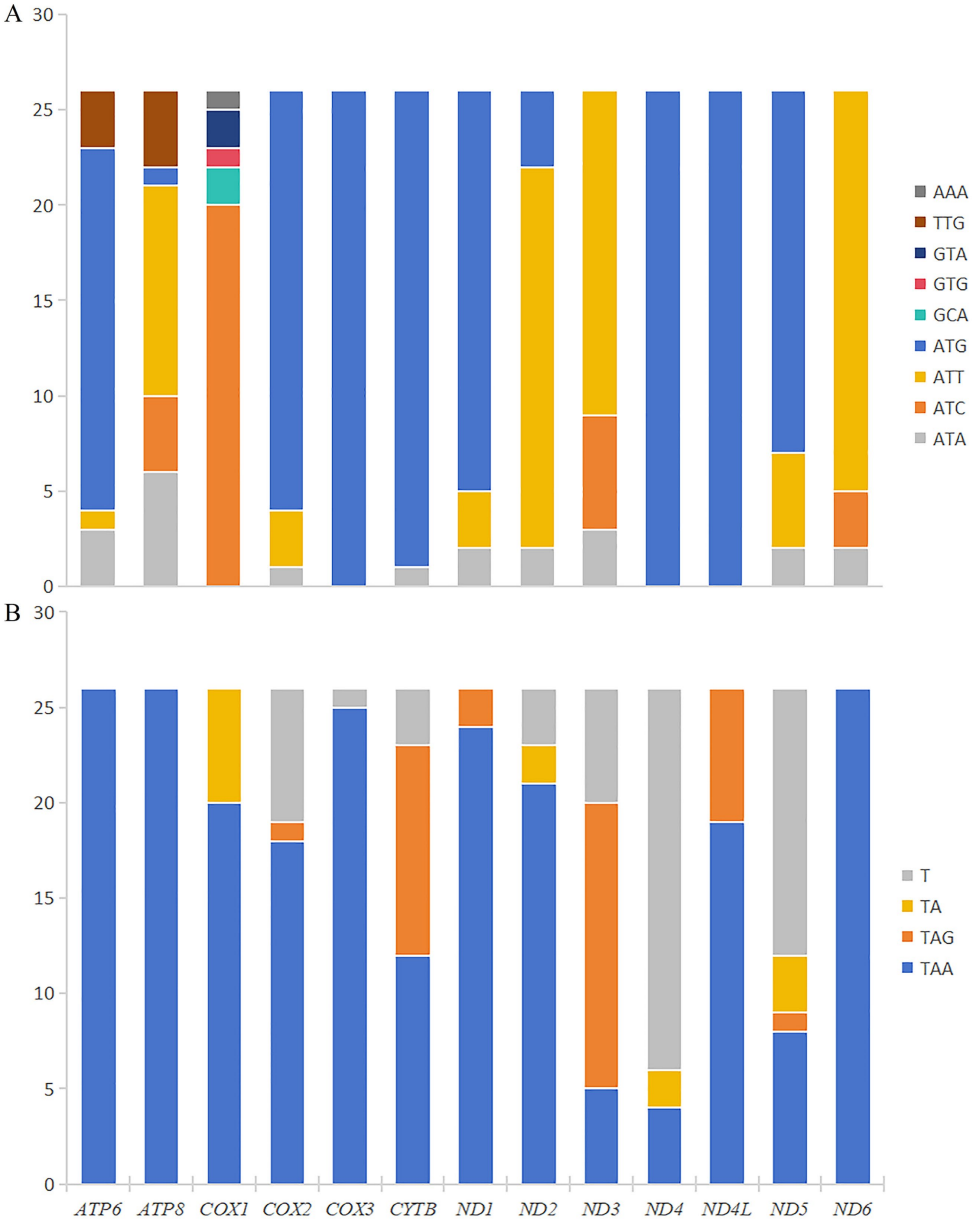
Comparison of start codons **(A)** and stop codons **(B)** of PCGs of 26 species.

The total length of 13 PCGs in *M. euteles* and *C. tesquorum sungaris* was 11,114 bp and 11,030 bp, respectively. Notably, *C. tesquorum sungaris* exhibited the shortest PCGs among the 26 flea species analyzed. The AT content within the PCGs of all examined flea species was significantly high, with *Xenopsylla cheopis* displaying the highest level at 80.31%. The AT-skew of PCGs ranged from −0.1557 to −0.1174, indicating a consistent negative trend. Conversely, GC-skew varied between −0.0060 and 0.0440, remaining positive except for *Neopsylla specialis* and *L. segnis* (Table 2). Analysis of RSCU in *M. euteles* and *C. tesquorum sungaris* revealed 27 and 26 high-frequency codons (RSCU>1), respectively. In both species, UUA was the most frequently used codon, while the least frequently used codons were CGC in *M. euteles* and UCG in *C. tesquorum sungaris* (Figure 1). Within the order Siphonaptera, the majority of PCGs utilized the canonical ATN as their start codon, with ATG being the most prevalent. Specifically, all 26 flea species examined used ATG as the initiating codon for *cox3*, *nad4*, and *nad4l*. In contrast, nonstandard start codons such as AAA, GCA, GTG, and GTA were identified in *cox1*, while TTG was observed in *atp6* and *atp8* (Figure 2A). Regarding stop codons, TAA was predominantly utilized by the 13 PCGs (Figure 2B).

**Figure 3:**
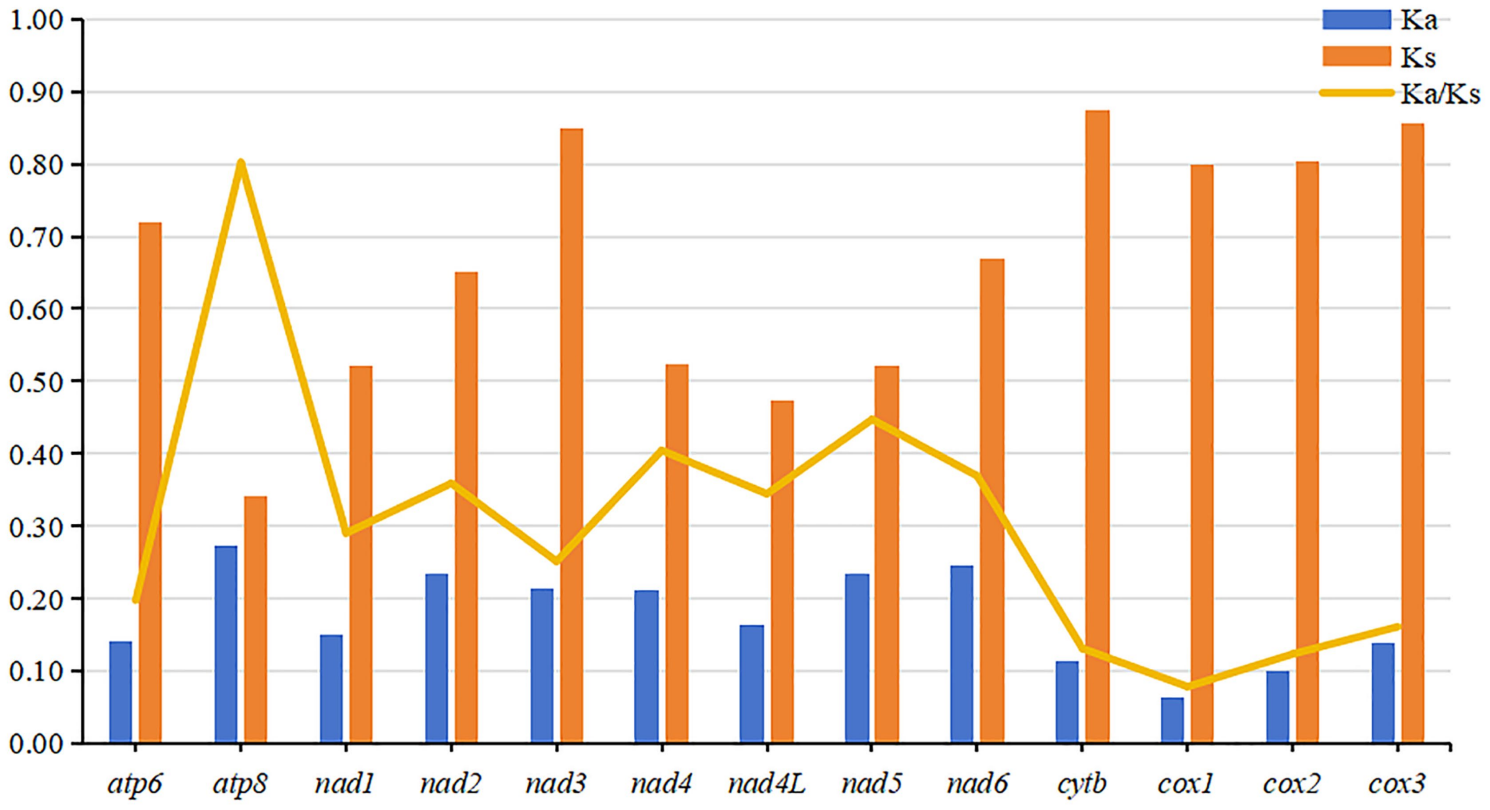
The evolutionary rates, represented by the ratio of nonsynonymous to synonymous substitutions (Ka/Ks), of 13 mitochondrial protein-coding genes (PCGs) across 26 flea species.

**Figure 4:**
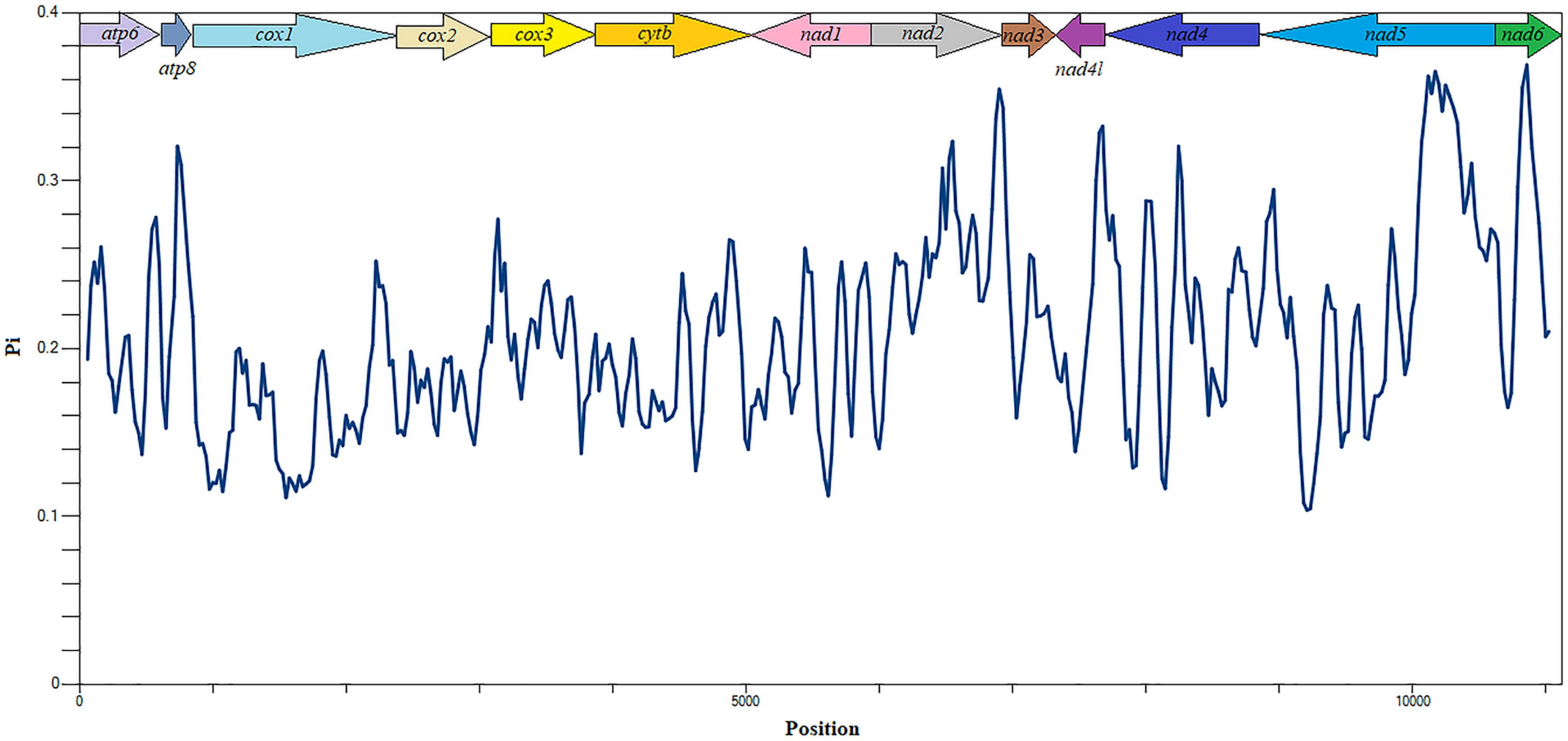
Nucleotide diversity analysis of 13 PCGs in Siphonaptera mitochondrial genomes.

The evolutionary rates and nucleotide diversity of 13 PCGs in the mitochondria of 26 flea species were evaluated through the analysis of Ka/Ks ratios and Pi values. The findings indicated that the average Ka/Ks ratios for each PCG ranged from 0.079 to 0.802, with all values remaining below 1, thereby suggesting that these genes have experienced purifying selection throughout their evolutionary history. Notably, *atp8* exhibited the highest rate of evolution, reflecting a relatively low intensity of purifying selection pressure. In contrast, *cox1* displayed the lowest mean Ka/Ks ratio and underwent significant purifying selection (Figure 3). Significantly higher nucleotide sequence variability (Pi values > 0.30) was observed in *atp8* and *nad2* genes, while markedly lower sequence variability (Pi values < 0.18) was noted in *cox1* and *cox2* genes (Figure 4).

### 3.3 Transfer RNA and ribosomal RNA genes

We performed a comprehensive analysis of 22 tRNA genes from the mitogenomes of 26 species. Our observations revealed that the sizes of these tRNAs ranged from 54 to 74 bp, with total lengths of tRNA genes varying between 1,405 and 1,439 bp, and AT content fluctuating between 78.84 and 81.12% (Table 2). Among the analyzed tRNA genes, 14 were encoded by the H strand, while the remaining 8 were encoded by the L strand.

The ribosomal RNA (rRNA) genes include the large ribosomal subunit RNA (*rrnL*) and the small ribosomal subunit RNA (*rrnS*). In all 26 flea species, the *rrnL* genes was located between the leucine transfer RNA (tRNA-Leu, *trnL1* (UAG)) and valine transfer RNA (tRNA-Val, *trnV*), with a size ranging from 1,196 to 1,317 bp. The AT content varied from 80.43 to 83.76%. Conversely, the *rrnS* gene was situated between the trnV gene and control region, measuring between 774 and 799 bp, with AT content ranging from 78.97 to 82.94% (Table 2).

### 3.4 Phylogenetic inference

**Figure 5:**
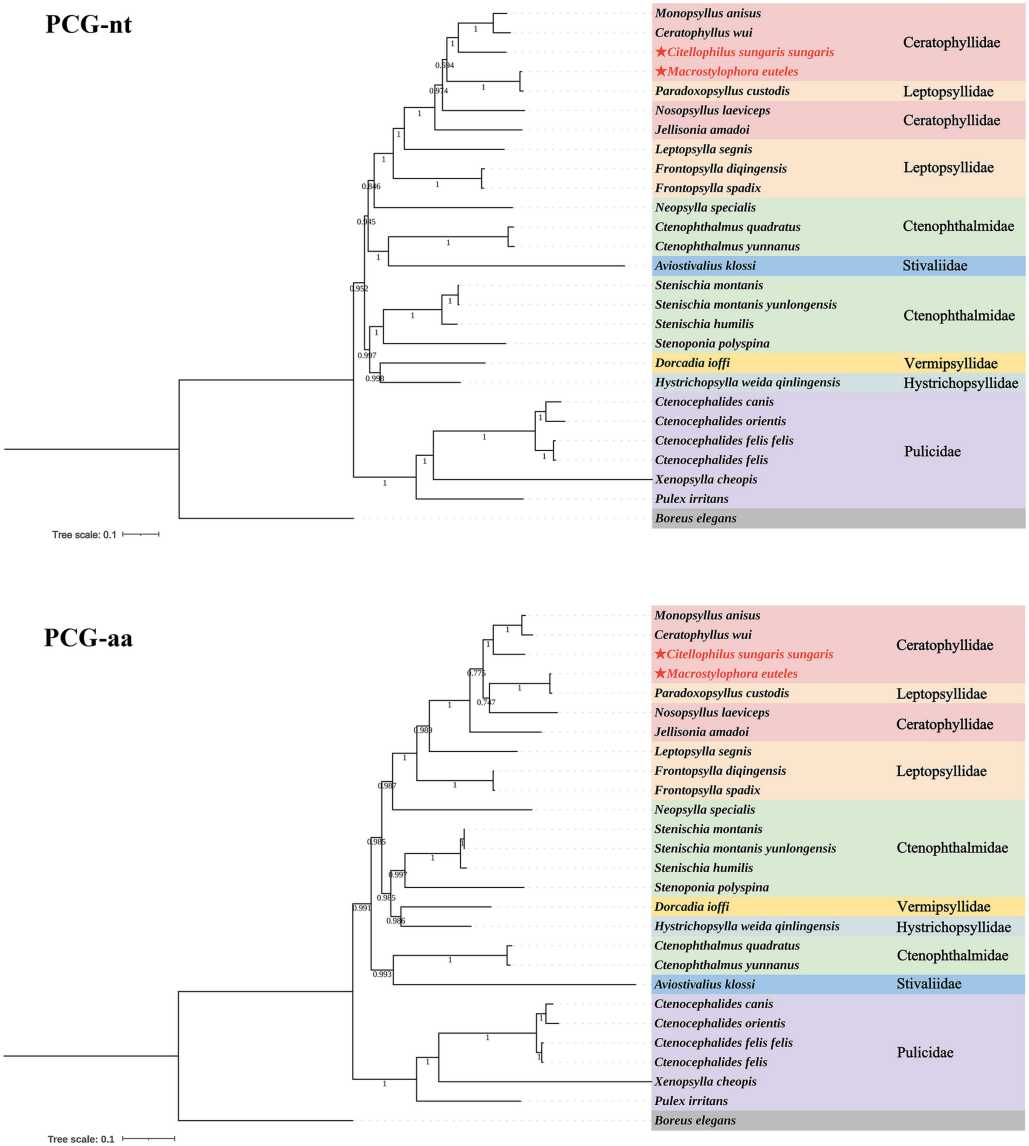
Phylogenetic trees for 26 species within the order Siphonaptera were constructed utilizing the Bayesian inference (BI) method.

**Figure 6:**
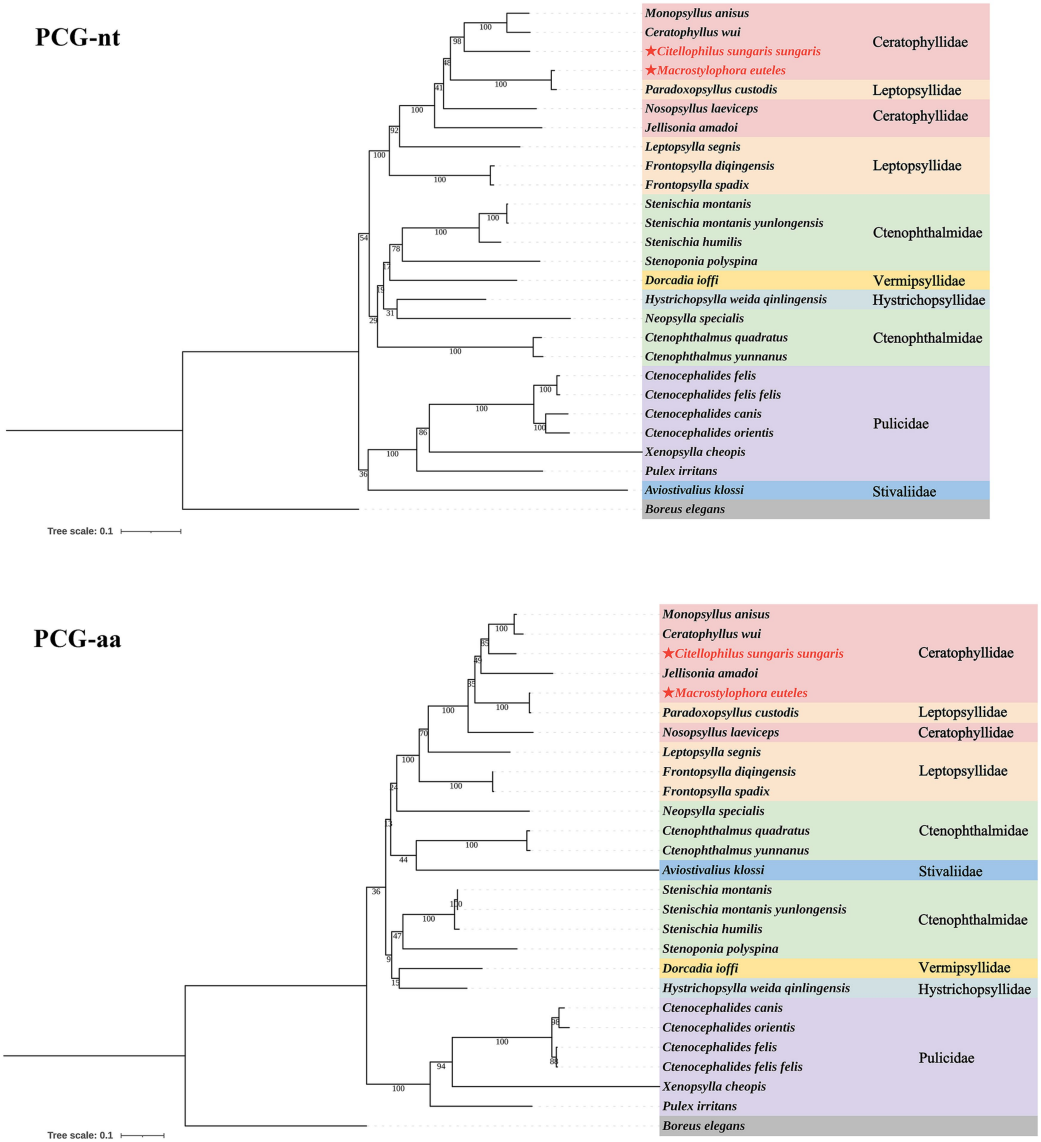
Phylogenetic trees for 26 species within the order Siphonaptera were constructed utilizing the maximum likelihood (ML) method.

Based on the nucleotide and amino acid sequences of 13 PCGs from 26 flea species across seven families within the order Siphonaptera, phylogenetic analysis using BI and ML methods generated four distinct tree topologies (Figures 5, 6). In the amino acid tree, all families were divided into two primary clades. The first clade comprised six families (Bpp = 0.991, Bv = 36): Ceratophyllidae, Leptopsyllidae, Ctenophthalmidae, Hystrichopsyllidae, Stivaliidae and Vermipsyllidae. The second clade consisted solely of Pulicidae (Bpp = 1, Bv = 100). Notably, in the nucleotide tree, Stivaliidae clustered with Pulicidae, but this arrangement was only supported in the ML tree (Bv = 36). Across all phylogenetic trees, the superfamilies Ceratophylloidea and Pulicoidea, as well as the family Pulicidae, consistently exhibited strong monophyly with support values of 100 in the ML analysis and 1 in the BI analysis. Conversely, the superfamily Hystrichopsylloidea, along with the families Ceratophyllidae, Leptopsyllidae and Ctenophthalmidae, were identified as paraphyletic. However, due to the limited representation of species within the families Pygiopsyllidae, Hystrichopsyllidae, and Vermipsyllidae (each containing only one species), their monophyly could not be conclusively determined. The four topologies indicated that *M. euteles* was most closely related to *Paradoxopsyllus custodis* (PCGnt: Bpp = 1, Bv = 100; PCGaa: Bpp = 1; Bv = 100), while *C. tesquorum sungaris* was closely associated with (*Monopsyllus anisus* + *Ceratophyllus wui*) (PCGnt: Bpp = 1, Bv = 98; PCGaa: Bpp = 1, Bv = 85). Additionally, the insertion *P. custodis* contributed to the paraphyly observed in the family Ceratophyllidae.

## 4 Discussion

The precise identification of fleas is a critical component in the study of these parasites and the diagnosis of flea-borne diseases. In this study, we sequenced the mitogenomes of *M. euteles* and *C. tesquorum sungaris*. By integrating data from the NCBI database, we conducted a comparative analysis of the mitogenomes of 26 flea species to elucidate the characteristics of Siphonaptera mitogenomes. The gene order in the flea mitogenome is conserved and aligns with that of the presumed ancestral mitogenome of insects (7). Among 26 flea species, the proportion of AT bases in their mitogenomes exceeds that of GC bases, indicating a pronounced AT bias. In Mecopterida, the sequences of Siphonaptera exhibited a higher AT content compared to those of Diptera and Mecoptera (45). The AT skew values were negative across all species, with the exception of *L. segnis* (AT-skew = 0.0236) and *Neopsylla specialis* (AT-skew = 0.0001). Similarly, the GC skew values were negative for all species except *L. segnis* (GC-skew = 0.2477). This observed strand bias could be attributed to asymmetric mutation pressures during DNA replication and/or transcription, possibly involving the deamination of adenine and cytosine nucleotides (15).

ATN is regarded as the predominant start codon in the class Insecta. In this study, ATN was identified as the most frequent start codon, whereas TTG was observed in *atp6* and *atp8*, and other rare start codons were found in *cox1*. Common stop codons in Siphonaptera are TAA and TAG; however, some species utilize incomplete stop codons such as T or TA, which can be converted to the complete stop codon TAA through polyadenylation post-transcription (1). The ratio of non-synonymous substitution rate (Ka) to synonymous substitution rate (Ks) exceeded 1, suggesting that the associated protein-coding genes were subject to positive selection. This positive selection pressure enabled the species to continually enhance its environmental adaptability. A Ka/Ks ratio of 1 indicates that protein-coding genes evolved neutrally, unaffected by natural selection. Conversely, a Ka/Ks ratio less than 1 signifies that protein-coding genes are under negative selection, which serves to constrain mitochondrial gene mutations, ensuring the stable function of proteins involved in mitochondrial oxidative phosphorylation (16). The Ka/Ks ratios for all 13 protein-coding genes were below 1, indicating that these genes maintained functional stability across the 26 flea species during evolution.

Evident inconsistencies were observed in the treatment of superfamily relationships across several previously published taxonomic classifications (24, 31–33, 44). This study confirmed the monophyly of the superfamilies Ceratophylloidea and Pulicoidea, whereas the superfamily Hystrichopsylloidea was found to be paraphyletic. These findings are consistent with a recent phylogenetic analysis based on five molecular markers (58) as well as mitochondrial genome data (5, 26). Whiting et al. (52) conducted the first comprehensive analysis of fleas using a four-locus molecular matrix, supporting the monophyly of the families Ceratophyllidae and Pulicidae. In contrast, Leptopsyllidae, Hystrichopsyllidae, and Ctenophthalmidae were identified as forming paraphyletic assemblages. Recent phylogenetic analyses employing maximum likelihood methods and utilizing five molecular markers (*18S rDNA*, *28S rDNA*, *EF-1α*, *cox1*, and *cox2*) indicated that the families Leptopsyllidae, Ceratophyllidae, Pulicidae, and Pygiopsyllidae form monophyletic groups, whereas the family Ctenophthalmidae was found to be paraphyletic (58). However, most recent phylogenetic studies based on mitochondrial data of Siphonaptera suggest that the family Ceratophyllidae may be paraphyletic (12, 26), consistent with the findings reported herein. It should be noted that the current study encompasses only approximately 1% of known flea species. To comprehensively evaluate the phylogenetic relationships within the order Siphonaptera and achieve more robust conclusions, it is imperative to decode a greater number of mitochondrial genomes from this order.

## 5 Conclusion

In this study, we conducted the first comprehensive analysis of the mitochondrial genomes of *Macrostylophora euteles* and *Citellophilus tesquorum sungaris*. The phylogenetic tree constructed based on the mitochondrial genome dataset exhibited distinct topology compared to previous studies, indicating the necessity for additional genomic data to elucidate the phylogeny of fleas more accurately. This research will provide valuable molecular data to support taxonomic and phylogenetic studies of fleas.

## Data Availability

All data and materials of the study are included in the manuscript and the Supplementary material. The complete mitochondrial genome data of *M. euteles* and *C. tesquorum* sungaris have been stored on the NCBI website under the accession number OR774969 and PP418872.

## Footnotes

^1^ http://mitos.bioinf.uni-leipzig.de/index.py

^2^ https://paulstothard.github.io/cgview/

^3^ http://tree.bio.ed.ac.uk/software/Figtree/
