# Supplementary material for: The complete mitochondrial genomes of Macrostylophora euteles and Citellophilus tesquorum sungaris and the phylogenetics of known Siphonaptera mitogenomes
Source: Front Vet Sci. 2025 May 1;12:1558328. doi: 10.3389/fvets.2025.1558328 (PMC12078284; doi:10.3389/fvets.2025.1558328)
Supplement: Supplementary file 1 [file Data_Sheet_1.DOCX]

Supplementary Material

# Supplementary Figures and Tables

## Supplementary Figures


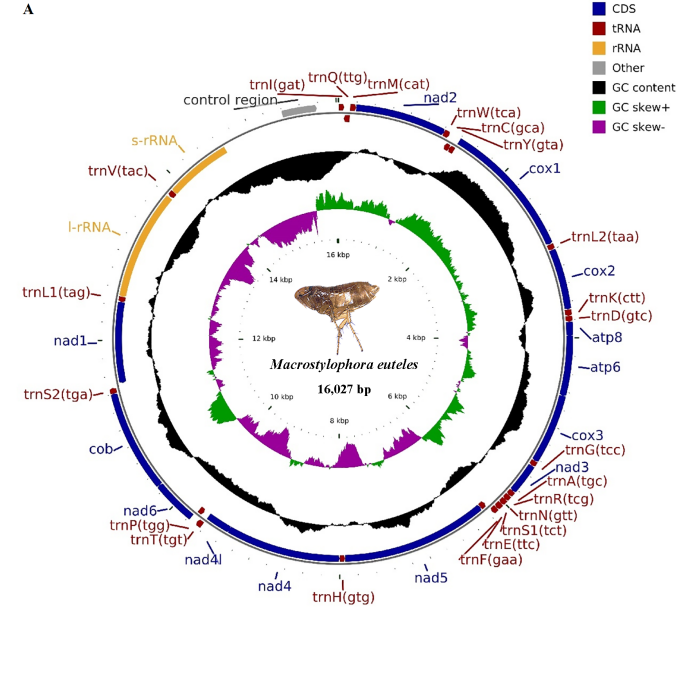

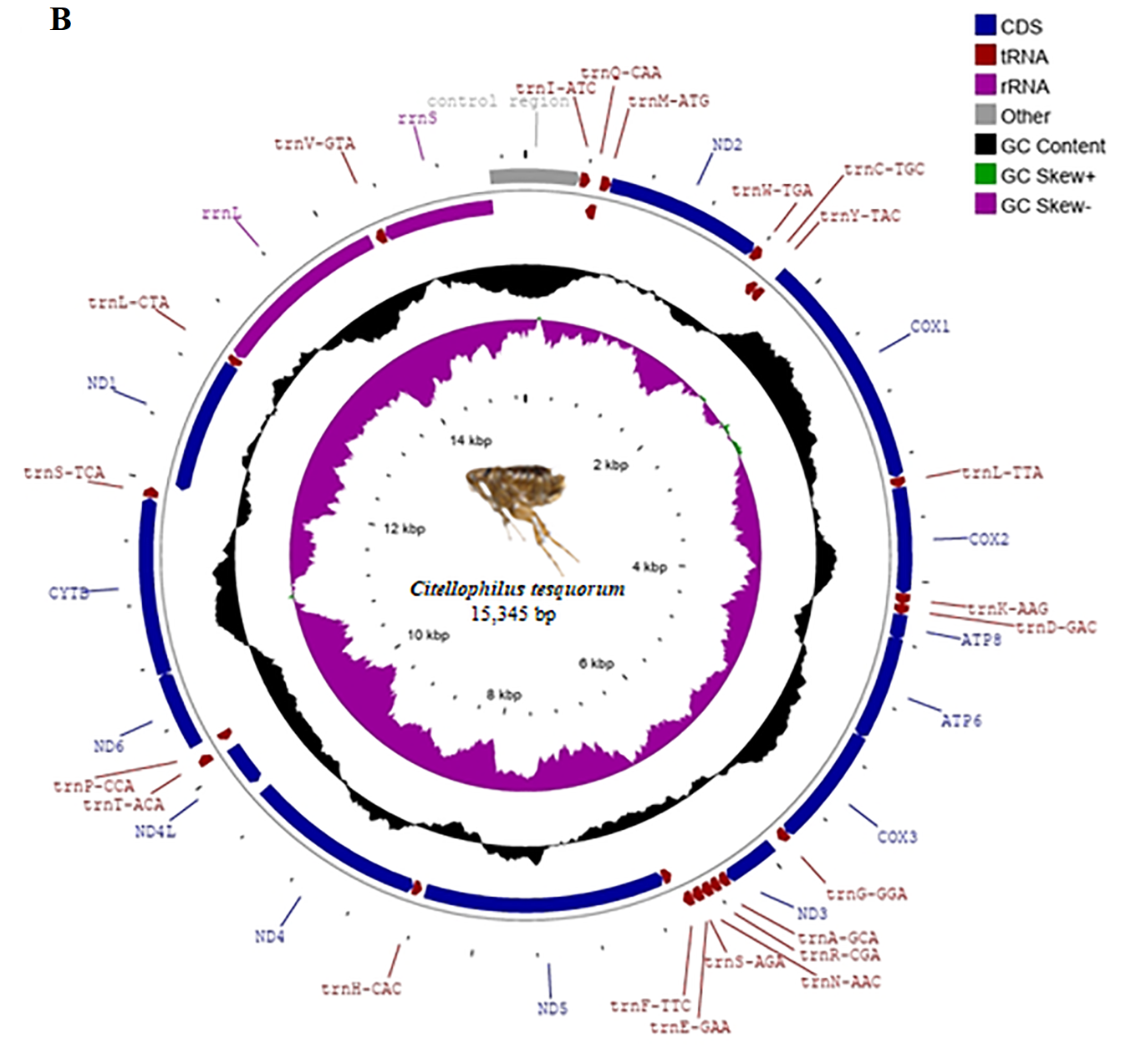


**Supplementary Figure 1.** The complete mitogenome maps of *Macrostylophora euteles* (A) and *Citellophilus tesquorum sungaris* (B).

- 1. **Supplementary Tables**

**Supplementary Table 1.** Organization of *Macrostylophora euteles* and *Citellophilus tesquorum sungaris* mitogenomes.

| **Gene** | **Strand** | **Position** | **Size(bp)** | **Initiation**  **Codon** | **Stop Codon** | **Anticodon** | **Intergenic**  **nucleotide** |
| --- | --- | --- | --- | --- | --- | --- | --- |
| trnI | H | 1-63/356-419 | 63/64 |  |  | GAT | -3/1 |
| trnQ | L | 61-129/421-489 | 69/69 |  |  | TTG | -1/-1 |
| trnM | H | 129-195/489-556 | 67/68 |  |  | CAT | 0/0 |
| nad2 | H | 196-1209/557-1570 | 1014/1014 | ATT/ATT | TAA/TAA |  | -2/-1 |
| trnW | H | 1208-1274/1570-1635 | 67/66 |  |  | TCA | 5/37 |
| trnC | L | 1280-1340/1673-1733 | 61/61 |  |  | GCA | 0/0 |
| trnY | L | 1341-1403/1734-1797 | 63/64 |  |  | GTA | -3/-3 |
| cox1 | H | 1401-2936/1795-3330 | 1536/1536 | ATC/ATC | TAA/TAA |  | 4/4 |
| trnL2 | H | 2941-3004/3335-3398 | 64/64 |  |  | TAA | 1/1 |
| cox2 | H | 3006-3686/3400-4080 | 681/681 | ATG/ATG | TAA/TAA |  | 2/2 |
| trnK | H | 3689-3758/4083-4152 | 70/70 |  |  | CTT | 0/0 |
| trnD | H | 3759-3823/4153-4217 | 65/65 |  |  | GTC | 0/0 |
| atp8 | H | 3824-3979/4218-4379 | 156/162 | ATT/ATT | TAA/TAA |  | -7/-7 |
| atp6 | H | 3973-4647/4373-5047 | 675/675 | ATG/ATG | TAA/TAA |  | -1/-1 |
| cox3 | H | 4647-5429/5047-5829 | 783/783 | ATG/ATG | TAA/TAA |  | 0/0 |
| trnG | H | 5430-5492/5830-5893 | 63/64 |  |  | TCC | 0/62 |
| nad3 | H | 5493-5843/5956-6306 | 351/351 | ATC/ATT | TAG/TAG |  | -2/-2 |
| trnA | H | 5842-5905/6305-6367 | 64/63 |  |  | TGC | 0/2 |
| trnR | H | 5906-5968/6370-6431 | 63/62 |  |  | TCG | -3/-3 |
| trnN | H | 5966-6030/6429-6494 | 65/66 |  |  | GTT | 0/0 |
| trnS1 | H | 6031-6098/6495-6562 | 68/68 |  |  | TCT | 0/0 |
| trnE | H | 6099-6164/6563-6626 | 66/64 |  |  | TTC | -2/-2 |
| trnF | L | 6163-6228/6625-6689 | 66/65 |  |  | GAA | 0/0 |
| nad5 | L | 6229-7936/6690-8379 | 1708/1690 | ATG/ATT | T/T |  | 1/28 |
| trnH | L | 7938-7999/8408-8470 | 62/63 |  |  | GTG | 0/0 |
| nad4 | L | 8000-9335/8471-9725 | 1336/1255 | ATG/ATG | T/T |  | -7/74 |
| nad4L | L | 9329-9622/9800-10093 | 294/294 | ATG/ATG | TAA/TAA |  | 2/2 |
| trnT | H | 9625-9692/10096-10159 | 68/64 |  |  | TGT | 0/0 |
| trnP | L | 9693-9755/10160-10223 | 63/64 |  |  | TGG | 17/11 |
| nad6 | H | 9773-10273/10235-10741 | 501/507 | ATT/ATT | TAA/TAA |  | -1/-1 |
| cytb | H | 10273-11409/10741-11880 | 1137/1140 | ATG/ATG | TAG/TAA |  | -2/6 |
| trnS2 | H | 11408-11471/11887-11951 | 64/65 |  |  | TGA | 30/16 |
| nad1 | L | 11502-12443/11968-12909 | 942/942 | ATG/ATG | TAA/TAA |  | 1/1 |
| trnL1 | L | 12445-12506/12911-12972 | 62/62 |  |  | TAG | 0/9 |
| rrnL | L | 12507-13793/12982-14229 | 1287/1248 |  |  |  | 12/40 |
| trnV | L | 13806-13873/14270-14337 | 68/68 |  |  | TAC | -1/-1 |
| rrnS | L | 13873-14652/14337-15117 | 780/781 |  |  |  | 747/0 |
| D-loop |  | 15400-15786/1-355;15118-15345 | 387/355;228 |  |  |  | 240/0 |
